# Symmetric dimethylarginine in dogs with myxomatous mitral valve disease at various stages of disease severity

**DOI:** 10.1371/journal.pone.0238440

**Published:** 2020-09-01

**Authors:** Carlotta Valente, Carlo Guglielmini, Oriol Domenech, Barbara Contiero, Eric Zini, Helen Poser

**Affiliations:** 1 Department of Animal Medicine, Production and Health, University of Padua, Padova, Italy; 2 AniCura Istituto Veterinario Novara, Granozzo con Monticello (NO), Italy; 3 Clinic for Small Animal Internal Medicine, Vetsuisse Faculty, University of Zurich, Zurich, Switzerland; Scuola Superiore Sant’Anna, ITALY

## Abstract

Symmetric dimethylarginine (SDMA) is a serum biomarker of renal damage in dogs. Moreover, SDMA concentration is an independent predictor of development of severe heart failure (HF) in humans with cardiac disease. This study evaluates whether the serum concentration of SDMA in dogs with myxomatous mitral valve disease (MMVD) is influenced by the severity of heart disease, pulmonary hypertension (PH) and treatment of HF. A total of 99 client-owned dogs were included in this retrospective case-control study; 78 dogs were affected by MMVD and classified according to the American College of Veterinary Internal Medicine (ACVIM) guidelines, and 21 were healthy controls. For each dog, history, physical examination, complete blood count, biochemical profile, thoracic radiography, 6-lead standard electrocardiogram and trans-thoracic echocardiography were available. Comparisons were performed between groups of dogs belonging to different ACVIM stages and between dogs with and without PH. The median SDMA concentration was neither significantly different among groups of dogs in different disease stages (overall P = 0.010), nor among dogs with MMVD, nor between those with [14.5 μg/dl (10.5–18.8)] and without PH [13 μg/dl (9–17.2)] (P = 0.295). The concentration of SDMA did not differ between dogs when considering the combined effect of the ACVIM group and cardiac treatment (overall P = 0.486). Furthermore, no correlation was found between SDMA concentration and radiographic and echocardiographic parameters associated with increased MMVD severity. In conclusion, this study failed to demonstrate the presence of renal impairment in dogs with MMVD, and the increase in renal parameters in some dogs in the more advanced stage of MMVD could be attributed to pre-renal azotemia.

## Introduction

Myxomatous mitral valve disease (MMVD) is the most common acquired heart disease and a frequent cause of heart failure (HF) in dogs [1]. Disease progression can be associated with an increase in left atrial pressure, the development of HF and/or pulmonary hypertension (PH) [2, 3]. According to the American College of Veterinary Internal Medicine (ACVIM) guidelines, four main stages of MMVD severity have been identified based on clinical, radiographic and echocardiographic parameters. These guidelines provide the treatment modalities for each stage [4, 5]. Stages A and B1 include dogs without any radiographic or echocardiographic signs of cardiac remodeling, for which no administration of cardiac drugs but only periodic monitoring is recommended. The stage B2 includes dogs with left atrial and/or left ventricular enlargement but without clinical signs of congestive HF that are or have been present in dogs in stage C and D. The administration of certain cardiac drugs, such as pimobendan and spironolactone, has clinical usefulness for dogs in stage B2, but the administration of different drugs, including diuretics, angiotensin-converting enzyme inhibitors (ACE-I), pimobendan and spironolactone is strongly recommended for dogs in stage C and D [6–11]. Before starting and pursuing a long-term diuretic therapy, renal function should always be assessed by measuring renal parameters and electrolyte concentrations [12]. Indeed, renal dysfunction is common in older dogs such as those affected by MMVD [13]. In humans, the concomitant renal and cardiac dysfunction, whereby the dysfunction of one organ induces dysfunction of the other, is defined as cardiorenal syndrome (CRS) [14]. This condition has also gained interest in veterinary medicine and in 2015 a consensus group published the definition and classification of CRS in dogs and cats [15].

Chronic kidney disease (CKD) has a prevalence of about 60% in people with CHF [16]. In dogs, the prevalence of CKD seems higher in those affected by MMVD compared to the aged-matched population [17], and the severity of renal impairment increases with the severity of heart disease [13]. Renal disorders are associated with a poor prognosis in their later stages, thus making their early diagnosis extremely important for treatment and to prevent progression [18]. According to the International Renal Interest Society (IRIS), serum creatinine (sCr) and symmetric dimethylarginine (SDMA) are the standard biomarkers used to diagnose CKD and grade its severity in dogs [19].

SDMA is a product of arginine methylation and is mainly excreted by the kidneys [20]. This parameter has been validated in dogs and cats, and is now routinely available [21, 22]. SDMA is inversely correlated with glomerular filtration rate (GFR) in both humans and dogs and is a valuable biomarker for early detection of kidney failure [23]. Moreover, its plasma concentration increases earlier than that of sCr and is less affected by extra-renal factors (e.g., age, body weight and muscular mass) [24–26].

In humans, SDMA gradually increases with the progression of heart disease and is an independent predictor of the development of severe HF; moreover, it is associated with a worse outcome and mortality [27]. In dogs with MMVD, two studies evaluated SDMA as a biomarker of heart disease severity using an “old” clinical classification [28] or the less recent and stringent ACVIM guidelines [29]. Hence, the aim of this study was to determine whether the serum concentration of SDMA in dogs with MMVD is influenced by stage of heart disease, presence of PH and/or the administration of drugs used to control the clinical signs of CHF.

## Material and methods

### Animals

This was a two centers retrospective study. Dogs were selected from the database of the cardiology units of the Veterinary Teaching Hospital of the University of Padua and the AniCura Istituto Veterinario Novara, between January 2014 and December 2016. Dogs with MMVD were enrolled if a complete clinical record was available including history, physical examination, complete blood count and biochemical profile. Diagnosis and staging of MMVD were obtained after carrying out a 6-lead standard electrocardiogram, standard thoracic radiography and complete trans-thoracic echocardiography.

The severity of the MMVD was assessed according to ACVIM guidelines. In particular, asymptomatic dogs without radiographic or echocardiographic evidence of cardiac remodeling were considered to be in stage B1; asymptomatic dogs with signs of cardiac remodeling [i.e., cardiomegaly with left atrium (LA) and/or left ventricle (LV) enlargement] were considered to be in stage B2; symptomatic dogs in which at least one episode of pulmonary edema and/or pleural effusion due to HF had occurred were considered to be in stage C, while symptomatic dogs with end-stage disease and refractory to standard therapies were considered to be in stage D [4, 5].

During the same period, dogs undergoing a cardiological evaluation as part of a general screening or pre-anesthesia evaluation for routine surgery (i.e., neutering or castration) and with normal results of physical examination, complete blood count, serum biochemical profile, electrocardiographic, thoracic radiographic and echocardiographic evaluation were selected as controls (group H). Dogs with incomplete clinical records, congenital or acquired heart disease other than MMVD, severe systemic concomitant disease and previous history of CKD were not included.

### Clinical pathology

Blood analyses [including complete blood count, alkaline phosphatase, bilirubin, aspartate aminotransferase, lactate dehydrogenases, glutamate dehydrogenases, glucose, sCr, blood urea nitrogen (BUN), albumin, total proteins, cholesterol, triglycerides, C-reactive proteins, calcium, phosphate, magnesium, sodium, chloride, potassium] were performed using automated analyzers for hematology (Advia 120 Hematology system, Siemens, Milan, Italy; Sysmex Xt 2000, IDEXX Laboratories) and biochemistry (BT 1500 Biotecnica, Roma Italy; AU680 Beckman, IDEXX Laboratories).

Aliquots of serum were frozen and sent to a commercial laboratory (IDEXX Laboratories) for SDMA determination using a high-throughput immunoassay.

### Cardiac imaging

Two radiographic orthogonal views of the thorax (right or left lateral and dorso-ventral or ventro-dorsal) were obtained for every dog included. The vertebral heart score was calculated from the lateral view as previously described [30].

Transthoracic echocardiography was performed from the right and left parasternal windows using standard projections by three experienced operators (OD, CG, HP) [31] with commercial ultrasound units (CX50, Philips, Eindhoven, Netherlands and Vivid-I, GE Healthcare, Milan, Italy). A simultaneous ECG trace was recorded during each exam. The M-mode interrogation of the LV was performed using the right parasternal short axis view, at the tip of the papillary muscles, and the measurements were performed using the leading edge to leading edge method [32]. The end-diastolic measurements were taken at the onset of the QRS complex on the ECG, while the end-systolic measurements were carried out at the point with the minimal distance between the LV free wall and the inter-ventricular septum (IVS). The end diastolic LV internal diameter (LVIDd) was normalized to the body weight (N-LVIDd) according to the allometric equation [33]. Left ventricular enlargement was considered for values of N-LVIDd ≥ 1.7. The left atrium (LA) to aorta (Ao) ratio was determined using the right parasternal short axis view at the heart base level as previously described [34] and LA enlargement was noted when LA/Ao ≥1.6. Mitral and tricuspid valve interrogation was performed using the left apical view and the corresponding diastolic blood flow was interrogated by pulsed wave Doppler with the sample gate positioned on the ventricular side, at the tip of the opened valvular leaflets. The early and late diastolic peak velocities of the trans-mitral blood flow were measured (MV E and MV A, respectively) and the MV E/A ratio was calculated.

The systolic right atrio-ventricular pressure gradient (TRPG) was calculated from the tricuspid regurgitation (TR) peak velocity (Vmax) using the modified Bernoulli equation. In the absence of RV outflow tract obstruction, values of TR Vmax > 3 m/s (TRPG > 36 mmHg) were considered indicative of pulmonary hypertension (PH). All the considered echocardiographic parameters were measured three times and averaged values were statistically analyzed.

### Statistical analysis

The dataset was analyzed using commercial statistical software (SAS, version 9.3, SAS Institute Inc., Cary, NC, USA). The distribution of data was assessed using the Shapiro-Wilk test. Statistical differences among the ACVIM and healthy groups and between dogs with and without PH were analyzed using an ANOVA model for the normally distributed variables, and the results were reported as Least Square Means (LSMEAN) with 95% confidence intervals (CI). For the purpose of the present study the ACVIM stage defined the allocation group of each dog (e.g., dogs with MMVD in ACVIM stage B1 were in group B1, dogs in ACVIM stage B2 were in group B2, etc.). Since few animals had refractory HF, for statistical purposes dogs in groups C and D were grouped together (C+D). The model included the fixed effects of the center of investigation, sex and age. Data not meeting the assumption of normality (i.e., sCr, BUN) were log-transformed and the results were reported as back transformed Least Square Means with 95% CI. When normality was not achieved despite transformation, variables were analyzed using the Kruskal-Wallis or Mann-Whitney tests and reported as median and interquartile range (IQR). Post-hoc pairwise comparisons among levels were performed using Bonferroni correction. The effect of treatment, and the combined effect of treatment and of the ACVIM stage on serum SDMA concentration were tested using the Kruskal-Wallis test. Linear regression between SDMA concentration and furosemide dose was assessed and the coefficient of determination (R2) was calculated. Correlations between SDMA concentration and demographic, clinical pathology, radiographic and echocardiographic parameters were assessed using the Spearman ranks correlation coefficient (r). Results were considered significant for P<0.05; for Spearman indexes, significance was set at P<0.001 provided that r was >0.50. Assuming P = 0.05 as the general significance level, for pairwise comparisons a value of P<0.008 was the threshold for every comparison.

## Results

### Study population

Ninety-nine dogs were included in the study (Table 1). Among them, 56 dogs (56.6%) were male and 43 (43.4%) female. The average age was 10.5 years (9.7–11.2) and the median (IQR) body weight was 8.5 kg (6.1–13.6). Of the included dogs, 50 (50.5%) were mongrels and 49 (49.5%) were pure-bred, comprising 5 (5%) Dachshund, 4 (4%) Jack Russell Terrier, 3 (3%) each of Cavalier King Charles Spaniel, Cocker Spaniel, German Shepherd and Labrador Retriever, and 2 (2%) Miniature Poodle, Beagle, Maltese, Boxer, Pug, Chihuahua, Pinscher and Shih-tzu; other breeds were represented by 1 dog each. Group H included 21 clinically healthy dogs. Among them, 7 dogs (33.3%) were male and 14 (66.7%) female, with a mean age of 7.6 years (6.0–9.1) and median BW of 12 kg (5.6–19). These dogs were significantly younger compared to those affected by MMVD in groups B1, B2 and C+D (P<0.005, P<0.001 and P<0.001, respectively).

**Table 1:** Clinical data of the 99 dogs included in the study.

Parameter	Total	H	B1	B2	C+D	Overall P
N. of dogs	99	21	37	17	24	
Sex M/F	56/43	7/14	22/15	13/4	14/10	
Age (Y)^†^	10.5 (9.7–11.2)	7.6 (6.0–9.1)	10.4 (9.2–11.5)*	12.0 (10.3–13.7)**	12.2 (10.8–13.7)**	<0.001
BW (Kg)^††^	8.5 (6.1–13.6)	12.0 (5.6–19)	8.5 (5.8–15.0)	8.6 (7.0–12.3)	7.3 (6.3–9.5)	0.420
Breed (N. of dogs)	Mongrel (50)		Mongrel (14)			
	Dachshund (5)					
	Jack Russell Ter. (4)					
	CKCS, Cocker					
	Spaniel, German	Mongrel (8)	Dachshund, Labrador			
	Shepherd, Labrador	Beagle (2)	Retriever (3)	Mongrel (11)	Mongrel (17)	
	Retriever (3)	Other breeds (11)	CKCS, Maltese, Cocker spaniel, German	Other breeds (6)	Jack Russell (2)	
	Min. Poodle, Beagle,		Shepherd (2)		Other breeds (5)	
	Maltese, Boxer, Pug,		Other breeds (9)			
	Chihuahua, Pinscher,					
	Shih-tzu (2)					
	Other breeds (12)					
Treatment						
N. of dogs (%)	34 (34)	1 (5)	2 (5)	10 (59)	21 (88)	
Loop diuretics	29 (29)	-	2 (5)	7 (41)	20 (83)	
ACE inhibitors	27 (27)	1 (5)	2 (5)	8 (47)	16 (66)	
Spironolactone	13 (13)	-	-	3 (18)	10 (42)	
Pimobendan	18 (18)	1 (5)	1 (3)	4 (24)	12 (50)	
Others	5 (5)	-	1 (3)	1 (6)	3 (13)	

Data are expressed as mean (95%CI) or median (interquartile range), for normally or not-normally distributed data.

^†^Normally distributed data were analyzed with ANOVA approach.

^††^Not-normally distributed data were analyzed with Kruskal-Wallis non parametric test.

*P<0.005 in comparison with H.

**P<0.001 in comparison with H.

Abbreviations: N, number; H, clinically healthy dogs; B1, dogs with myxomatous mitral valve disease (MMVD) belonging to B1 stage of severity according to ACVIM guidelines; B2, dogs with MMVD belonging to B2 stage of severity according to ACVIM guidelines; C+D, dogs with MMVD belonging to C and D stage of severity according to ACVIM guidelines; M/F, male/female; BW, body weight; CKCS, Cavalier King Charles Spaniel.

Of the 99 dogs, 78 (78.8%) were affected by MMVD at different ACVIM stages. Among these, 37 (47.4%), 17 (21.8%) and 24 (30.8%) dogs were classified as B1, B2 and C+D, respectively. Fifty-six dogs (56.6%) had TR and PH was diagnosed in 20 of them (35.7%), whereas PH status could not be determined in the remaining 43 dogs (43.4%) because of the absence of TR.

Thirty-three dogs (42.3%) with MMVD were receiving cardiovascular treatments, while 45 dogs (57.7%) were treatment-naïve at the time of examination (Table 1). Among the treated dogs, 8 (24.2%), 10 (30.3%) and 15 (45.4%) were receiving one, two and three or more cardiovascular drugs, respectively (Table 2).

**Table 2:** Cardiovascular drugs (single or in combination) received by dogs with myxomatous mitral valve disease grouped according to the disease severity.

	Total	B1	B2	C+D
**Monotherapy, N. of dogs (%)**	8 (24.2%)			
ACE-I, N. of dogs (%)	2 (6%)	1 (3%)	1 (3%)	
Loop diuretics, N. of dogs (%)	6 (18.2%)		1 (3%)	5 (15.2%)
**Combination of 2 drugs, N. of dogs (%)**	10 (30.3%)			
Pimo + Loop diuretics, N. of dogs (%)	1 (3%)	1 (3%)		
ACE-I + Loop diuretics, N. of dogs (%)	7 (21.2%)	1 (3%)	2 (6%)	4 (12.1%)
Pimo + ACE-I, N. of dogs (%)	1 (3%)			1 (3%)
ACE-I + Spiro, N. of dogs (%)	1 (3%)		1 (3%)	
**Combination of 3 drugs, N. of dogs (%)**	3 (9%)			
Pimo + ACE-I + Loop diuretics, N. of dogs (%)	3 (9%)		2 (6%)	1 (3%)
**Combination of 4 drugs, N. of dogs (%)**	12 (36.4%)			
Pimo + ACE-I + Loop diuretics + Spiro, N. of dogs (%)	12 (36.4%)		2 (6%)	10 (30.3%)
**Total**	33 (100%)	3 (9.1%)	9 (27.3%)	21 (63.6%)

Abbreviations: N, number; B1, dogs with myxomatous mitral valve disease (MMVD) belonging to B1 stage of severity according to ACVIM guidelines; B2, dogs with MMVD belonging to B2 stage of severity according to ACVIM guidelines; C+D, dogs with MMVD belonging to C and D stage of severity according to ACVIM guidelines; ACE-I, angiotensin converting enzyme inhibitor; Pimo, pimobendan; Spiro, spironolactone.

### Laboratory and cardiac parameters

The serum concentration of SDMA was above the upper limit of the reference range in 2 (9.5%), 7 (18.9%), 3 (17.6%) and 10 (41.7%) dogs in group H, B1, B2 and C+D, respectively; sCr was above the upper limit of the reference range in 2 dogs in the H and B2 groups and in only 1 dog in the B1 and C+D groups, while BUN was above the upper limit of the reference range in 1 dog in groups H and B1 and in 4 dogs in group C+D. Both sCr and BUN concentrations were concomitantly above the upper limit of the reference range in 3 (8.1%), 2 (11.8%) and 9 (37.5%) dogs in group B1, B2 and C+D, respectively. Among animals with a concomitant increase in sCr and BUN concentrations, 3 in group B1, 1 in group B2 and all 9 in group C+D also had increased SDMA, whereas 1 dog in group B2 with a concomitant increase in sCr and BUN had a normal SDMA concentration.

**Fig 1:**
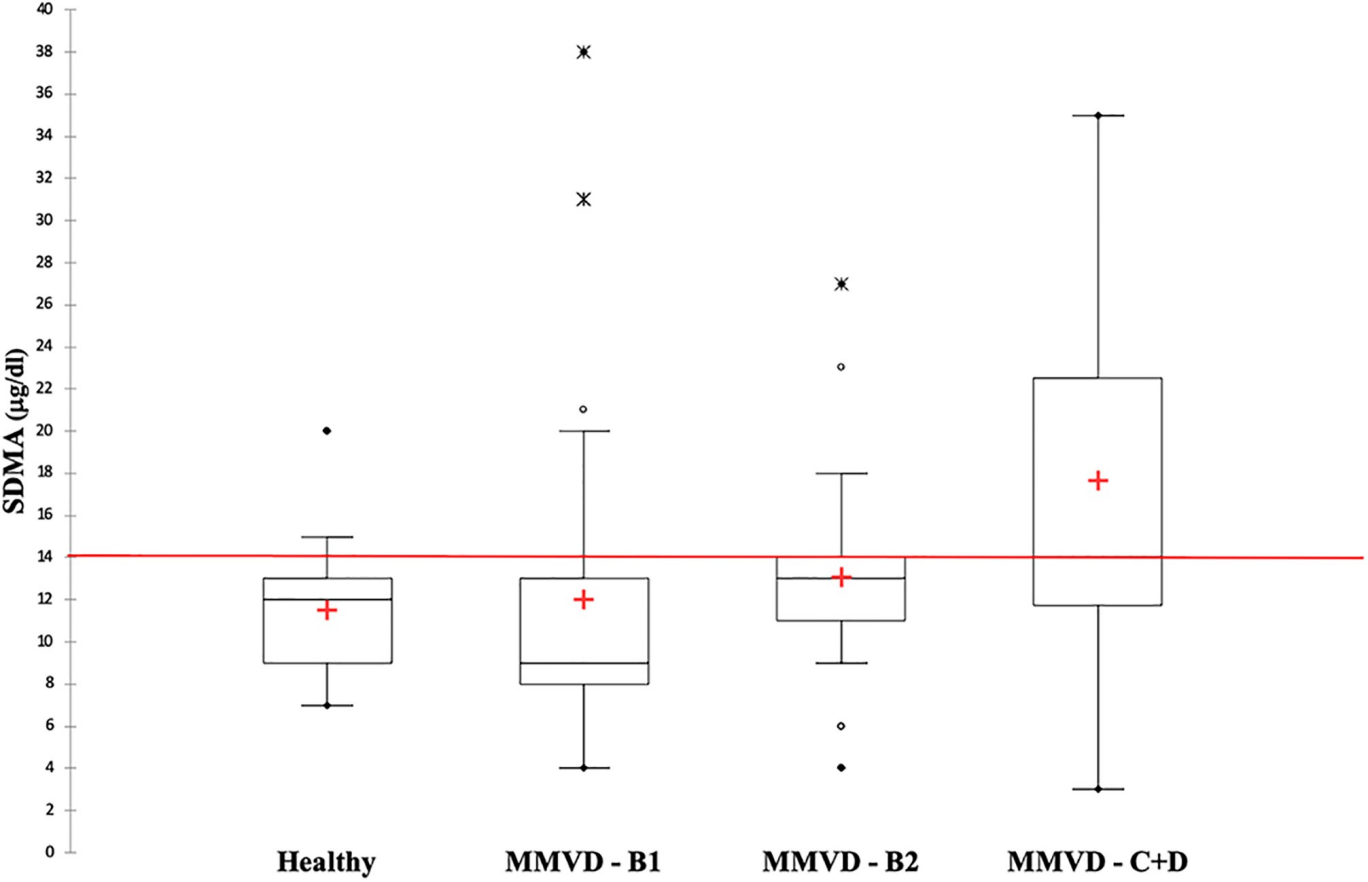
Box and whisker plot of symmetric dimethylarginine (SDMA) concentration in 21 clinically healthy dogs (H) and in 78 dogs with myxomatous mitral valve disease (MMVD) grouped according to the ACVIM classification. The red horizontal line represents the reference values of SDMA, full circles the minimum and maximum values, the cross the median values, open circles the outliers and the asterisks the extreme outliers.

The median SDMA concentration did not differ significantly among groups (overall P = 0.010). The mean BUN concentration was significantly higher in group C+D [49.4 mg/dl (39.9–61.1)] compared to that of group H [22.4 mg/dl (18–28.1)] and B1 [27.6 mg/dl (23.3–32.8)] (P<0.001 for both comparisons). The mean sCr concentration was significantly higher in group C+D [1.2 mg/dl (1.0–1.4)] compared with group B1 [0.8 mg/dl (0.7–0.9)] (P = 0.007) (Table 3 and Fig 1).

**Table 3:** Clinical pathology, radiographic and echocardiographic parameters of 21 clinically healthy dogs (H) and 78 dogs with myxomatous mitral valve disease grouped according to disease stages (B1, B2, C+D).

Parameter	H	B1	B2	C+D	Overall P
BUN (mg/dl)^1^	22.4 (18–28.1)	27.6 (23.3–32.8)	33.9 (26.4–43.7)	49.4 (39.9–61.1)**^,^°°	<0.001
sCr (mg/dl)^1^	0.9 (0.7–1.0)	0.8 (0.7–0.9)	1.0 (0.8–1.2)	1.2 (1.0–1.4)°	0.009
Phos (mg/dl)^†^	3.5 (3.0–4.1)	3.0 (2.5–3.4)	3.1 (2.4–3.7)	3.4 (2.9–3.9)	0.354
SDMA (μg/dl)^††^	12.0 (9.0–13.0)	9.0 (8.0–13.0)	13.0 (11.0–14.0)	14.0 (11.8–22.5)	0.010
Na+ (mEq/l)^†^	147.4 (145.0–149.8)	148.2 (146.3–150.0)	150.2 (147.4–153.0)	150.9 (148.5–153.3)	0.138
K+(mEq/l)^†^	4.4 (4.1–4.7)	4.8 (4.6–5.0)	4.8 (4.4–5.1)	4.5 (4.2–4.8)	0.129
VHS^†^	10.7 (10.0–11.4)	10.4 (9.9–11.0)	11.8 (11.2–12.4)°	12.6 (12.1–13.1)**^,^°°	<0.001
LA/Ao^††^	1.5 (1.4–1.5)	1.5 (1.4–1.5)	2.1 (1.9–2.6)**^,^°°	2.6 (2.2–3.3)**^,^°°	<0.001
N-LVIDd^†^	1.4 (1.3–1.6)	1.5 (1.4–1.6)	2.0 (1.9–2.1)**^,^°°	2.1 (1.9–2.2)**^,^°°	<0.001
MV E (cm/s) ^†^	67.1 (57.4–76.9)	65.2 (57.7–72.7)	114.8 (103.5–126.0)**^,^°°	134.5 (125.1–143.8)**^,^°°	<0.001
MV A (cm/s) ^†^	44.6 (34.1–55.1)	66.0 (57.9–74.1)	80.6 (68.5–92.7)**	86.6 (75.3–97.8)**	<0.001
MV E/A^†^	1.3 (1.1–1.6)	1.0 (0.8–1.2)	1.5 (1.3–1.8)	1.8 (1.6–2.0)°°	<0.001
TR Vmax (m/s) ^†^	NA	2.6 (2.3–2.8)	3.1 (2.8–3.4)	3.1 (2.9–3.4)°	0.003
TRPG (mmHg) ^†^	NA	26.8 (20.9–32.7)	40.4 (32.6–48.2)	41.0 (34.6–47.2)°	0.003

Data are expressed as mean (95% CI) or median (interquartile range), for normally or not-normally distributed data.

^†^Normally distributed data analyzed with ANOVA approach.

^††^Not-normally distributed data analyzed with Kruskal-Wallis non parametric test.

^1^ for back-transformed data, LS Means were reported with 95% CI.

* = P<0.008 with H;

** = P<0.001 with H.

° = P<0.008 with B1;

°° = P<0.001 with B1.

^§^ = P< 0.008 with B2;

^§§^ = P<0.001 with B2.

Abbreviations: H, clinically healthy dogs; B1, dogs with myxomatous mitral valve disease (MMVD) belonging to B1 stage of severity according to ACVIM guidelines; B2, dogs with MMVD belonging to B2 stage of severity according to ACVIM guidelines; C+D, dogs with MMVD belonging to C and D stage of severity according to ACVIM guidelines; BUN, blood urea nitrogen; sCr, serum creatinine, Phos, serum phosphorus; SDMA, symmetric dimethylarginine; VHS, vertebral heart score; LA/Ao, left atrium to aorta ratio; N-LVIDd, left ventricular internal end diastolic diameter normalized to the body weight; MV E, mitral valve early diastolic velocity; MV A, mitral valve late diastolic velocity; TR Vmax, tricuspid regurgitation peak velocity; NA, Not available; TRPG, tricuspid regurgitation pressure gradient.

In dogs with MMVD, there was no significant difference in serum SDMA concentration between those with and without PH (P = 0.295).

The serum SDMA concentration of dogs with MMVD was significantly and positively correlated with sCr (r = 0.58; 95% CI = 0.43–0.70; P<0.001) and BUN (r = 0.51; 95% CI = 0.34–0.64; P<0.001), but not with the other clinical and laboratory variables or radiographic and echocardiographic parameters (Table 4).

**Table 4:** Spearman’s ranks correlations index between symmetric dimethylarginine (SDMA) and demographic, clinical pathology, radiographic and echocardiographic parameters obtained from 21 healthy dogs and 78 dogs with myxomatous mitral valve disease at different disease stages.

Parameter	r	95% CI	P
Age	0.24	0.04–0.41	0.02
BW	-0.06	-0.25–0.14	0.57
BUN	**0.51**	**0.34–0.64**	**<0.001**
sCr	**0.58**	**0.43–0.70**	**<0.001**
Phos	0.004	-0.20–0.20	0.98
Na+	-0.002	-0.21–0.20	0.98
K+	-0.01	-0.21–0.20	0.94
VHS	0.34	0.09–0.55	0.007
LA/Ao	0.22	0.03–0.40	0.02
N-LVIDd	0.27	0.07–0.44	0.007
MV E	0.28	0.08–0.45	0.006
MV A	0.09	-0.11–0.29	0.37
MV E/A	0.14	-0.07–0.33	0.19
TR Vmax	0.15	-0.11–0.39	0.26
TRPG	0.15	-0.11–0.39	0.27

Significant correlations are evidenced in bold.

Abbreviations: BW, body weight; BUN, blood urea nitrogen; sCr, serum creatinine; Phos, serum phosphorus; VHS, vertebral heart score; LA/Ao, left atrium to aorta ratio; N-LVIDd, left ventricular internal end diastolic diameter normalized to the body weight; MV E, mitral valve early diastolic velocity; MV A, mitral valve late diastolic velocity; TR Vmax, tricuspid regurgitation peak velocity; TRPG, Tricuspid regurgitation pressure gradient.

**Fig 2:**
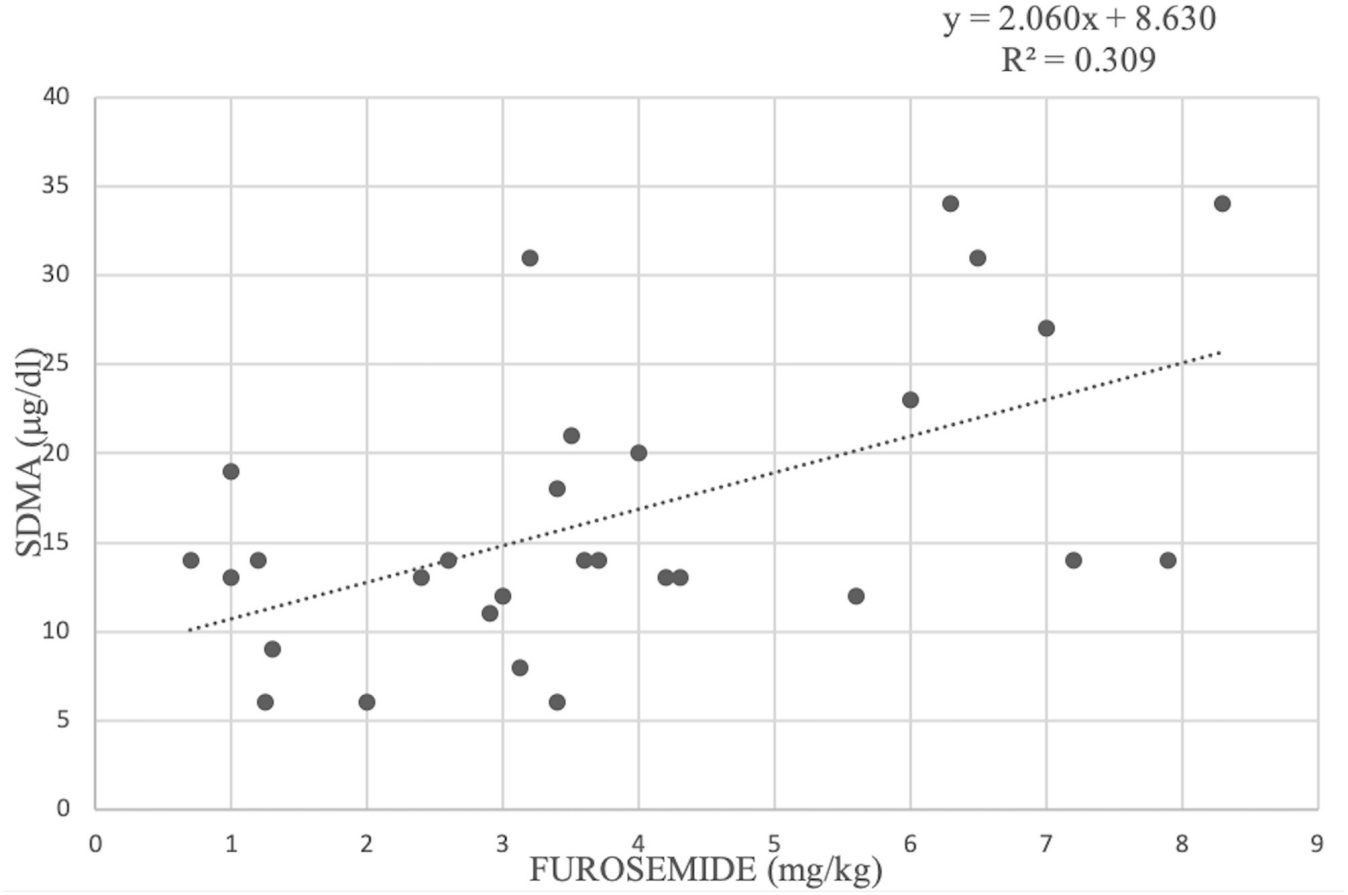
Linear regression between serum symmetric dimethylarginine (SDMA) concentration and furosemide dose in 29 dogs with myxomatous mitral valve disease. R^2^ represents the coefficient of determination of the model.

Regarding treatments, there was a positive correlation between furosemide dose and serum SDMA concentrations (R^2^ = 0.309) (Fig 2). The administration of one or more cardiac drugs, including pimobendan, ACE-I, furosemide and spironolactone, was associated with higher serum concentration of BUN, sCr and SDMA (P = 0.038; P = 0.013 and P = 0.026, respectively), in dogs under cardiac therapy compared to those that were not being treated (Table 5). However, when the analysis was repeated after combining cardiac treatment and ACVIM stages, no significant difference was found for the serum concentration of SDMA (P = 0.486) (Table 6).

**Table 5:** Values of renal clinical pathology parameters in dogs with myxomatous mitral valve disease treated or not with cardiovascular drugs.

Parameter	Not treated	Treated	*P-value*
BUN (mg/dl)	29.5 (18–45)	43 (24–64)	0.038
sCr (mg/dl)	0.8 (0.6–1.1)	1.0 (0.8–1.4)	0.013
SDMA (μg/dl)	10 (9–14)	14 (11–19)	0.026

Values are expressed as median (interquartile range).

Abbreviations: BUN, blood urea nitrogen; sCr, serum creatinine; SDMA, symmetric dimethylarginine.

**Table 6:** Combined effect of treatment and myxomatous mitral valve disease stage according to the American College of Veterinary Internal Medicine (ACVIM) classification on symmetric dimethylarginine (SDMA) concentration in dogs in ACVIM stage B2 and C.

ACVIM class	Not treated	Treated	*P-value*
**SDMA—B2** (μg/dl)	13 (11–14)	14 (6–14)	0.93
**SDMA–C** (μg/dl)	17 (3–35)	13.5 (11–20)	0.99

Mann-Whitney non parametric test.

Data are reported as median (interquartile range).

Abbreviations: SDMA, symmetric dimethylarginine.

## Discussion

The results of this study failed to demonstrate a significant increase in SDMA concentration with the progression of the disease stage in dogs with MMVD. Furthermore, SDMA concentration was positively correlated with sCr and BUN, parameters used to identify renal dysfunction, but not with radiographic and echocardiographic parameters associated with cardiac disease severity, such as left atrial and ventricular dilation or peak velocity of the MV E wave.

Our results were similar to those of another study conducted on a small sample of dogs with MMVD, where disease’s severity was classified according to first and less stringent ACVIM guidelines [29]. However, in a previous study conducted in dogs with MMVD, the SDMA concentration was positively correlated with the severity of HF as well as with radiographic vertebral heart score and echocardiographic indices of left atrial and ventricular dilation [28]. The discrepancy between the present and this latter study can be partially attributed to the use of different systems for staging the severity of MMVD. In the present study, the more recent ACVIM guidelines [5] were used, whereas in that previous study the severity of HF was assessed according to the International Small Animal Cardiac Health Council (ISACHC) classification. This classification was mainly based on the severity of clinical signs and, for instance, dogs presenting with pulmonary edema could be reallocated to a less severe stage of HF following appropriate therapy. Conversely, the ACVIM guidelines combine clinical, radiographic and echocardiographic findings for staging disease severity and affected dogs can only progress to more advanced stages of HF. The use of two diverse staging methods probably led to different groupings of dogs, thus explaining the difference between studies.

Moreover, in the present series the number of dogs in group D (three dogs) was too small to form a single group, and thus they were merged with those of group C. Two of the three dogs in group D had very high values of SDMA (i.e., 31 and 34 μg/dl) while the third dog had an SDMA concentration at the upper limit (14 μg/dl). These high values suggest that, in the more advanced stage of the disease, renal impairment may be present but these findings need further confirmation with a higher number of dogs.

There was no significant increase in SDMA in dogs with MMVD and PH compared to those without PH and no correlation was found between SDMA and TRPG, an echocardiographic index of PH severity. In dogs with MMVD, the presence of PH is associated with increased LA pressure and pulmonary venous congestion, which develops in more advanced stages [3]. Collectively, the lack of SDMA increase in the more advanced stages of cardiac disease along with the absence of correlation with radiographic and echocardiographic indices of left-sided cardiac remodeling suggest a poor link between SDMA and MMVD progression. However, because dogs with MMVD in this study had only mild or moderate PH, the association between PH and SDMA needs confirmation in a cohort of dogs with severe PH.

The serum concentration of BUN was significantly higher in dogs with MMVD in group C+D compared to those in group H and B1, whereas the sCr concentration was significantly higher in dogs in group C+D than in group B1. Moreover, all dogs in group C+D with a concomitant increase in both sCr and BUN also had an SDMA concentration above the upper reference range.

Dogs in ACVIM stages C or D need to receive diuretics such as furosemide or torasemide promptly, and this therapy is usually continued in the long-term [4, 5]. In these dogs diuretic therapy is expected to affect renal function, leading to an increased serum concentration of renal biomarker [17, 35]. Dehydration due to diuretic treatment can lead to pre-renal azotemia, as previously reported [13, 17, 36, 37], even if primary renal impairment cannot be avoided in some of these dogs.

The observed increase in SDMA concentration along with the increase in furosemide dosage confirms this hypothesis. The observed correlation between furosemide dosage and SDMA concentration has not been previously reported in dogs and suggests that the administration of diuretics might affect this parameter, as observed for the other kidney function tests [38]. After combining the administration of cardiac therapy and ACVIM staging the analysis yielded no significant effect of these parameters on SDMA concentration. This result can be explained by hypothesizing that the actual heart condition of dogs, rather than therapy itself, may have an influence on SDMA.

The present results indicate that the CRS of humans [14], namely a specifically reduced renal function as a consequence of cardiac disease, is less likely to occur in dogs. In particular, humans with acute or chronic heart disease associated with a reduced ejection fraction, such as acute myocardial infarction and coronary artery disease, develop a progressive reduction in renal perfusion and, in turn, a decrease in GFR; the decreased renal function is accompanied by an increase in SDMA [27, 39, 40]. The absence of a strong evidence of CRS in dogs with MMVD could be explained by the differences in the major pathophysiologic mechanisms. Indeed, compared with humans with HF, systolic dysfunction is not common in dogs with MMVD and, therefore, the cardiac output and renal perfusion of the latter are usually maintained with the only exception of the end-stage stages of MMVD [41].

Because of its retrospective design this study has some limitations. First, clinical information was not recorded in a standardized way for all dogs and the exclusion of pre-existing primary renal disease was not possible. The latter would have required additional diagnostics, including the determination of GFR, which is uncommonly performed in dogs with cardiac disease in a clinical setting. Second, the collection of cases was not consecutive and, thus, some selection bias can have occurred. Furthermore, the sample size was relatively small, particularly for dogs in the more advanced stage of MMVD, and the presence of a statistical type II error cannot be completely excluded. However, we calculated the sample size that was necessary to obtain a statistical power of 80% with alpha = 0.05 using either an a priori and post hoc approach based on results of a previous study [28] and of the present study, respectively. The calculated number of animals to be included in each group was 8 dogs and 23 dogs, respectively. Thus, the sample size of our groups fit completely and almost completely the criteria commonly used to calculate sample size.

Moreover, considering that healthy control dogs are younger than MMVD’s ones, an effect of the age on SDMA values can not be completely excluded.

Furthermore, the lack of correlation between SDMA concentration and echocardiographic parameters used to assess disease severity suggests that SDMA concentration and, therefore, renal function is poorly influenced by MMVD in dogs. Third, in people right heart catheterization is the gold standard technique for PH diagnosis; however, it is uncommonly used in dogs, while echocardiography is more often performed despite its intrinsic limitations [42]. Finally, the effect of previous treatment on SDMA concentration was evaluated by considering a multimodal approach but not for each single treatment.

## Conclusions

In conclusion, the results of this study failed to demonstrate that renal function, evaluated by measuring serum SDMA concentration, is significantly impaired in dogs with MMVD. Although some dogs in the C+D group of MMVD had an increased concentration of the variables used to identify renal dysfunction, this was most likely due to pre-renal azotemia instead of representing a feature of the CRS described in humans.

## Supporting information

S1 Dataset Data of all animals included in the study. (XLSX)
